# Production of Laccase by a New *Myrothecium verrucaria* MD-R-16 Isolated from Pigeon Pea [*Cajanus cajan* (L.) Millsp.] and its Application on Dye Decolorization

**DOI:** 10.3390/molecules22040673

**Published:** 2017-04-23

**Authors:** Jiao Sun, Na Guo, Li-Li Niu, Qing-Fang Wang, Yu-Ping Zang, Yuan-Gang Zu, Yu-Jie Fu

**Affiliations:** 1 Key Laboratory of Forest Plant Ecology, Ministry of Education, Northeast Forestry University, Harbin 150040, China; sunjiaoxu@163.com (J.S.); octo_9010@163.com (N.G.); niulili0613@126.com (L.-L.N.); 18846136923@163.com (Q.-F.W.); 18846135590@163.com (Y.-P.Z.); yuangangzu@163.com (Y.-G.Z.); 2 Engineering Research Center of Forest Bio-Preparation, Ministry of Education, Northeast Forestry University, Harbin 150040, China

**Keywords:** *Myrothecium verrucaria*, laccase, guaiacol, optimization, dye decolorization application

## Abstract

The present study was conducted to screen a laccase-producing fungal endophyte, optimize fermentation conditions, and evaluate the decolorization ability of the laccase. A new fungal endophyte capable of laccase-producing was firstly isolated from pigeon pea and identified as *Myrothecium verrucaria* based on a ITS-rRNA sequences analysis. Meanwhile, various fermentation parameters on the laccase production were optimized via response surface methodology (RSM). The optimal fermentation conditions were a fermentation time of five days, temperature 30 °C and pH 6.22. Laccase activity reached 16.52 ± 0.18 U/mL under the above conditions. Furthermore, the laccase showed effective decolorization capability toward synthetic dyes (Congo red, Methyl orange, Methyl red, and Crystal violet) in the presence of the redox mediator ABTS, with more than 70% of dyes decolorizing after 24 h of incubation. Additionally, the activity of laccase was relatively stable with pH (4.5–6.5) and a temperature range of 35–55 °C. Therefore, the high laccase production of the strain and the new fungal laccase could provide a promising alterative approach for industrial and environmental applications.

## 1. Introduction

Synthetic dyes have been widely utilized in many industries, such as paper, textiles, printing, cosmetics, and pharmaceuticals. This results in the generation of large amounts of highly polluted effluents [1,2]. The effluents containing highly toxic and potentially carcinogenic pollutants are released into the environment, which leads to serious environmental problems [3]. Several physicochemical methods were reported to remove dyes from waste water. But these methods suffer from a number of limitations such as high cost, low efficiency, and production of large amounts of toxic sludge, aromatic amines, and secondary waste [4,5]. Therefore, it is necessary to develop high-efficiency, eco-friendly, and low cost methods to remove dyes from waste water.

Laccase (benzenediol: oxygen oxidoreductase, EC 1.10.3.2), a member of enzymes known as the multi-copper containing oxidases, can catalyze oxidation of an array of substrates including mono-, di-, and polyphenols, methoxyphenols, and ascorbate via the simultaneous reduction of oxygen to water [6,7]. In the presence of certain low molecular weight compounds, named redox mediators, such as 2,2′-azino-bis(3-ethylbenzothiazoline-6-sulfonate) (ABTS) and 1-hydroxybenzotriazole (HBT), laccases are able to oxidize compounds that are not laccase substrates [8]. Currently laccases are extensively employed in pulping, bleaching, bioremediation, biosensor, food technological uses, and in the treatment of industrial wastewater [9,10,11,12]. Many studies have demonstrated that these laccases can decolorize dyes [4,10,13,14,15,16,17,18]. Thus, research concerning laccase is being carried out for removing dyes from industrial effluent, which is commonly considered to be lower-cost, more effective, and environmentally safe.

Laccases are widely distributed in plants, bacteria, insects and fungi [19]. In fungi, laccases are mainly produced by ascomycetes, basidiomycetes, and deuteromycetes [20,21]. Some fungi capable of producing laccases have been isolated and characterized from soil and wood sources [5,14,20,22], but most of laccases commonly have low enzymatic activity [6]. Furthermore, the laccase production generally requires a long fermentation time [23]. These performances limit their large-scale commercial and industrial applications. Therefore, exploitation of novel potential laccase producers with shorter fermentation time and higher enzyme activity is necessary to remove synthetic dyes.

In the present study, a novel fungal endophyte capable of laccase-producing was isolated and screened from pigeon pea [*Cajanus cajan* (L.) Millsp.]. Moreover, the fermentation conditions for laccase production by this fungal endophyte were optimized by central composite design (CCD). Furthermore, Congo red (CR), Methyl orange (MO), Methyl red (MR) and Crystal violet (CV) were used for investigating the decolorization effect by the laccase from this fungal endophyte. Additionally, MR was chosen as the model dye for investigating the effects of pH, temperature, and ABTS concentration on the decolorization ability of the laccase.

## 2. Results and Discussion

### 2.1. Isolation and Identification of Laccase-Producing Fungal Endophyte

**Figure 1:**
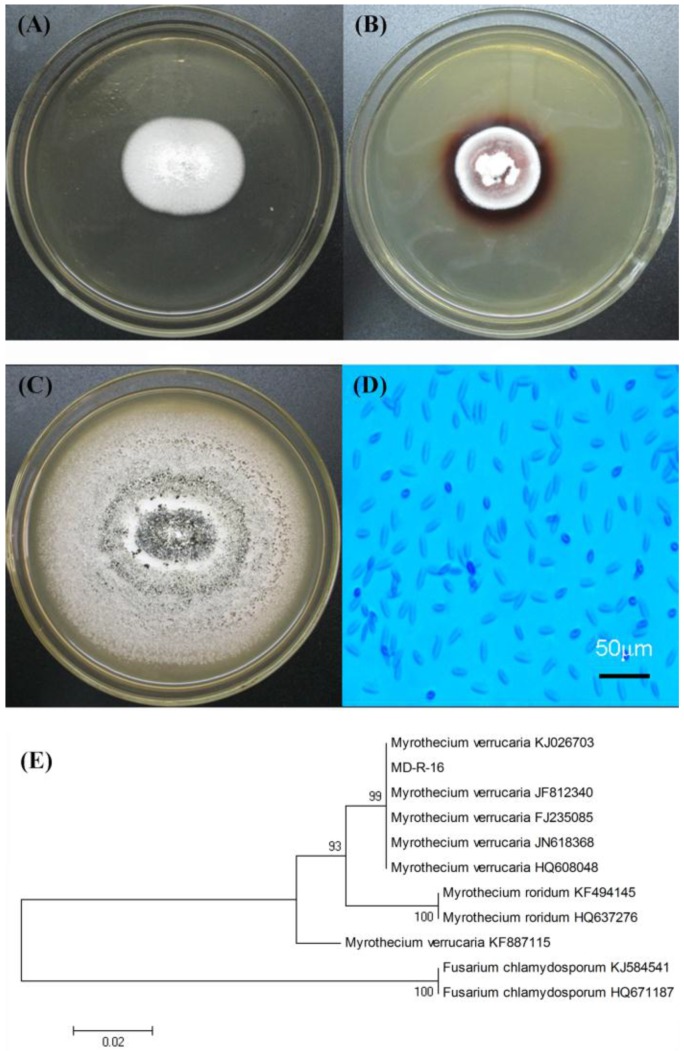
Laccase-producing fungal endophyte isolated from pigeon pea. (**A**) laccase-producing fungal endophyte on the potato dextrose agar (PDA) without guaiacol; (**B**) laccase-producing fungal endophyte on the PDA with laccase indicator-guaiacol; (**C**) and (**D**) represented the fungal endophyte MD-R-16 of colonial morphology and micrographic characteristics (×400), respectively; (**E**) phylogenetic tree constructed by the program neighbor-joining (NJ) based on ITS1-5.8S-ITS2 sequences of laccase-producing fungal endophyte. Bootstrap values (1000 tree interactions) are indicated at the nodes.

As demonstrated in Figure 1A,B, the fungal endophyte was found to produce a colored halo on the PDA plate containing guaiacol, as it was the oxidation-reduction reaction. The same phenomenon was found in some fungal laccase producers such as *Trichoderma harzianum* [21], *Paraconiothyrium variabile* [24], *Trametes* sp. LS-10C [25], and *Pseudolagarobasidium acaciicola* LA 1 [26]. According to these results, we inferred that the fungal endophyte may produce laccase or other oxidases such as peroxidases and bilirubin oxidase. The fungal endophyte that was probably capable of laccase-producing, was firstly isolated from roots of pigeon pea plants, named as MD-R-16 and were selected for further study. Figure 1C,D show the colonial morphology and micrographic characteristics of the fungal endophyte MD-R-16, respectively. The fungal endophyte MD-R-16 was white lanose and grown radially, but after several days of culturing, the color of the fungal endophyte MD-R-16 was variable from white to deep green. The shape of spores was fusiform or elliptical, and conidia were 8.0–50.0 μm × 2.0–5.0 μm. On the basis of the colony and spore morphological analysis, the fungal endophyte MD-R-16 was revealed to be closest to *Myrothecium verrucaria* belonging to deuteromycete [27].

To taxonomically identify the fungal endophyte MD-R-16, the ITS-rDNA genes were amplified through PCR. ITS have been generally used to examine relationships among various fungi, particularly between closely related species [28]. The amplified rDNA fragment was approximately 548 bp in length. Based on the phylogenetic analysis of the aligned ITS genes (Figure 1E), the fungal endophyte MD-R-16 exhibited 99% homology to *Myrothecium verrucaria* (99% of probability to several *M. verrucaria* strains: accession numbers KJ026703, JF812340, FJ235085, JN618368, and HQ608048). Sequence data has been submitted to GenBank under accession numbers JQ356542. Therefore, the fungal endophyte MD-R-16 was identified as *M. verrucaria*.

In order to eliminate the interference of peroxidases (lignin peroxidase and manganese peroxidase), we used the method descried by Adak et al. [26]. The results showed that there were no peroxidases in the crude enzymes of *M. verrucaria* MD-R-16, but the laccase activity or bilirubin oxidase activity was detected. Additionally, according to the method of Murao et al. [29] to detect the activity of bilirubin oxidase, the result indicated that the crude enzymes of *M. verrucaria* MD-R-16 was unable to oxidize bilirubin to biliverdin, so there was no bilirubin oxidase in the crude enzymes. What′s more, it is reported that some fungi belonging to *M. verrucaria* can produce laccases, such as *M. verrucaria* NF-05 [20], *M. verrucaria* 24G-4 [30], and *M. verrucaria* NF-08 [31]. On the basis of these, we conclude that *M. verrucaria* MD-R-16 can mainly produce laccase in this work.

### 2.2. Determination of Laccase Activity

**Figure 2:**
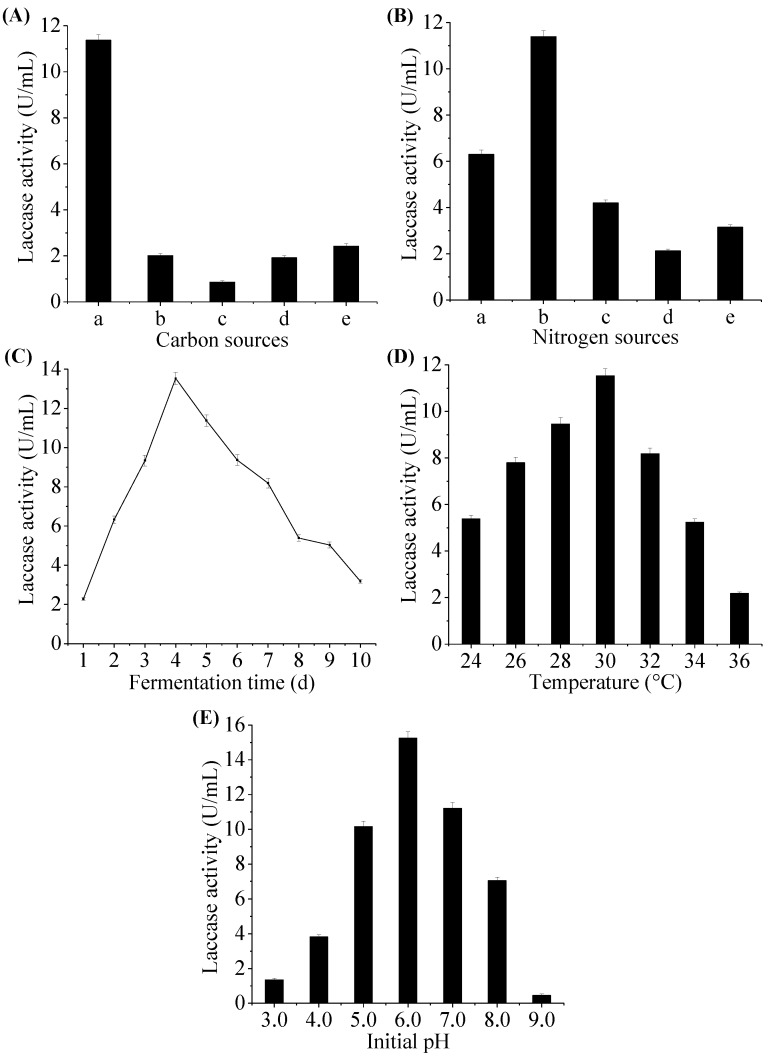
Effects of nutrient and fermentation factors on laccase production by *M. verrucaria* MD-R-16. (**A**) Effect of carbon sources on laccase production. The carbon source (from a–e) is successively glucose, sucrose, starch, lactose, and maltose. (**B**) Effect of nitrogen sources on laccase production. The nitrogen source (from a–e) is successively yeast extract, peptone, beef extract, ammonium chloride, and ammonium nitrate. (**C**) Effect of fermentation time on laccase production. (**D**) Effect of temperature on laccase production. (**E**) Effect of initial pH values on laccase production. All experiments are done by changing one independent variable while fixing others at certain levels.

Common factors have important effect on laccase production, such as carbon sources and nitrogen sources, as well as other factors, temperature, pH, and fermentation time [3,9], and these factors were primarily studied using the one single factor method in this work. As presented in Figure 2A, the laccase production achieved the highest value of 11.37 ± 0.26 U/mL, when glucose was used as the carbon source. Hao et al. [32] reported that glucose facilitated the highest production of laccase by *Pestalotiopsis* sp. Peptone was found to be the optimum nitrogen source for laccase production in Figure 2B. A similar finding was reported by Gao et al. [31], where peptone supported the maximum laccase production from *M. verrucaria* NF-08. Consequently, glucose and peptone were used as the carbon source and the nitrogen source in the following experiments, respectively.

Moreover, in order to improve laccase production, the different fermentation parameters, such as the initial pH, fermentation temperature and time, which play vital roles in the metabolic activity of microbial cell were examined. Figure 2C shows the laccase production by *M. verrucaria* MD-R-16 occurred during the first day and the laccase production achieved its highest value on the fourth day being 13.53 ± 0.22 U/mL. During the fermentation process, the nutrients in the medium could be gradually used up, which was able to affect the fungi physiology that led to the inactivation of secretary machinery of the enzymes. In that case, the induction of sporulation might explain a shorter incubation time with higher laccase activity [33]. Sadhasivam et al. [34] reported that the laccase produced by *T. harzianum* occurred on the second day and reached its maximum on the fourth day. A report on *Trametes hirusta* indicated the maximum laccase activity reached 7.614 U/mL after an incubation time of 20 days [35]. The maximum laccase activity reached 11.53 ± 0.12 U/mL at 30°C in Figure 2D. Further increase in temperature led to decrease in laccase production which is in congruence with other reports on *Trametes* sp. LS-10C [25], *Cotylidia pannosa* [33], and *Arthrospira maxima* [36]. In general, the fungi are cultivated at temperatures between 25°C and 30°C for optimal laccase production [37]. Results in Figure 2E indicated that an increase in laccase activity was observed with the increase of pH from 3.0 to 6.0. After that, there was a decrease in laccase activity. The maximum laccase activity of 15.26 ± 0.20 U/mL was obtained at pH 6.0. Abd El Monssef et al. [21] stated that pH between 4.5 and 6.0 is suitable for enzyme production.

### 2.3. Optimization of Fermentation Conditions for Laccase Production by Fungal Endophyte MD-R-16

Temperature, initial pH, and time play significant roles in the fungi fermentation, as these have been reported to influence laccase production by other microorganisms [9]. In this work, we found the initial pH, fermentation temperature, and time were three significant factors for laccase production from the above experiment. Therefore, CCD was applied to determine the optimal levels of the three selected factors. According to the experimental data from CCD (Table 1), a second order polynomial equation was obtained for laccase activity, as seen in the following equation (in terms of the actual factors):

(1) $$\begin{array}{l} Y=16.24+2.27X_{1}-0.75X_{2}+0.74X_{3}-0.60X_{1}X_{2}-0.34X_{1}X_{3}+0.66X_{2}X_{3}\\ -3.65X_{1}^{2}-1.99X_{2}^{2}-2.60X_{3}^{2} \end{array}$$

where, *Y* is the activity of laccase (U/mL); *X*_1_ is the fermentation time (d); *X*_2_ is the fermentation temperature (°C); and *X*_3_ is pH of the initial fermentation medium.

The results of ANOVA for the quadratic model were summarized in Table 2. The values of *R*^2^ and adjusted *R*^2^ were 0.9974 and 0.9950 respectively, which were desirable determination coefficients and had advocated a high correlation between the experimental and predicted values of the response. Meanwhile, a non-significant value of lack of fit (*p* > 0.05) and a highly significant level of the model (*p* < 0.01) were obtained by statistical analyses, suggesting that the mode could be accurately predicted by the variation [38]. Therefore, the model can be used for further analysis. As the results presented in Table 2, the factors with the largest effect on the laccase production were linear terms of *X*_1_, *X*_2_, and *X*_3_, interaction term of *X*_1_*X*_2_ and *X*_2_*X*_3_, and quadratic terms of *X*_1_^2^, *X*_2_^2^, and *X*_3_^2^.

**Figure 3:**
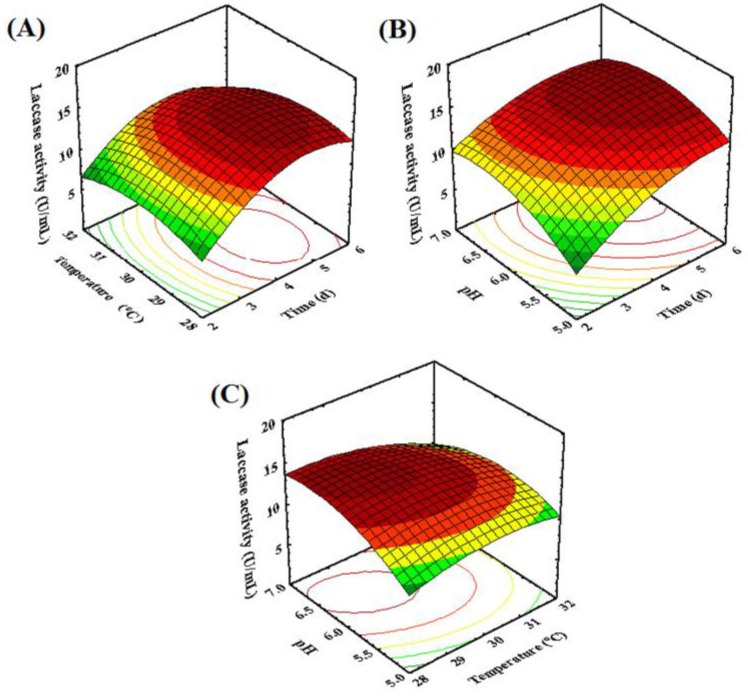
Response surfaces plots for the laccase production by *M. verrucaria* MD-R-16: (**A**) varying the fermentation temperature and time; (**B**) varying the fermentation time and pH of the initial fermentation medium; (**C**) varying the fermentation temperature and pH of the initial fermentation medium.

Figure 3 shows the three-dimensional response surface graphs representing the regression model for laccase production by the fungal endophyte MD-R-16, which demonstrated the interaction among three variables to determine the optimum level of each variable for maximum response. It can be observed from Figure 3A that the interactive effects of fermentation time and temperature significantly influenced the laccase production. The laccase production initially increased by increasing fermentation time and temperature, and then decreased with the longer fermentation time and higher fermentation temperature. This may be attributed to the fact that the nutrients in the medium could be gradually used up, which could affect the fungi physiology that led to the inactivation of the secretary machinery of enzymes. The isolated fungal endophyte exhibited a high laccase activity as cultivated no more than five days, much shorter than the previous study [39], so it can be a good candidate for the application in commerce.

As presented in Figure 3B, the laccase activity rose with the increase in pH value and fermentation time. While pH changed from 5 to 6.22, an increase of laccase activity was observed. There was a decrease in laccase activity when the pH was further increased. The lower pH condition could affect the fungal growth, which would inhibit the metabolism of laccase synthesis thus resulting in lower laccase production [22]. In addition, the condition with a low pH may alter the three-dimensional structure of the enzymes [40]. These results were consistent with the conclusions of some previous reports on the laccase production by other fungal species, such as *Fusarium solani* [22], *Pycnoporus sanguineus* [41], and *Ganoderma* sp. [42]. The results indicated that appropriate pH could improve the laccase production.

As shown in Figure 3C, the laccase production increased slightly with the increase of fermentation temperature at higher pH. In addition, Figure 2C also described that the laccase activity decreased significantly with the increase in temperature. Sporulation was induced with fermentation temperature increasing, which could hamper the mycelia growth in fungi. Hence, it might cause the decrease in enzyme activity. It was found that the enzyme activity was reduced when fungi were cultivated at temperatures higher than 30°C [25,43].

Based on the mathematical model, the optimal fermentation conditions of the laccase production by *M. verrucaria* MD-R-16 were obtained as follows: fermentation time 4.44 d, fermentation temperature 29.73 °C, and pH 6.22. Considering the actual operations, the fermentation time and temperature were modified as five days and 30 °C, respectively in the following experiments. In this condition, the estimated laccase activity was 16.52 ± 0.18 U/mL. The present result of higher laccase activity with a short fermentation period would be advantageous for application and commercialization.

### 2.4. Dye Decolorization Test

**Figure 4:**
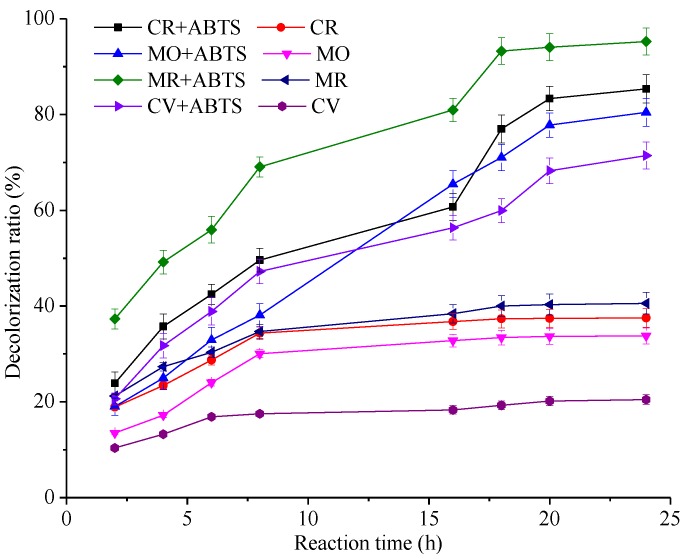
Effects of reaction time on the decolorization of different dyes (CR, MO, MR, CV) in laccase or an ABTS-laccase mediated system. Data were expressed as mean ± SD (n = 3).

Dye decolorization efficiency depends upon their chemical structures, enzymes, and system conditions. Figure 4 shows the effects of time course of decolorization on the selected dyes by the laccase, in the presence or the absence of ABTS. In the absence of ABTS, the laccase showed decolorization capability toward the selected dyes, with less than 50% of dyes decolorizing after 24 h of incubation, indicating that the laccase can decolorize the synthetic dyes. The reason is that fungi could produce some low-molecular weight compounds that can potentially act as a redox mediator [44]. In the presence of ABTS, our observations have suggested that the dye decolorization was continuously increased during the first 18 h and no obvious change was observed up to 24 h. The drop in the rate of dye decolorization after an initial fast reaction could be due to inactivation of the enzyme. Besides, the substrate of ABTS can be oxidized by laccase. Then ABTS becomes an active cation radical. However, in the presence of dyes, ABTS^+^ may take electrons from dyes and recover to their original state [15,45]. Thus, ABTS can be recycled in the process of decolorization [45]. With the dye decolorization, less and less electron-rich compounds can be used by ABTS^+^, which may lead to a slight change in the rate of decolorization.

As a representative azo dye, MR (50 mg/L) was rapidly decolorized to a maximum extent of 95.37% ± 1.03% after incubation for 24 h. It was also demonstrated that the laccase performed at a high decolorization capacity toward CR (50 mg/L) and MO (50 mg/L), with 85.37% ± 0.89% and 80.46% ± 0.99%, respectively after incubation for 24 h. Meanwhile, the laccase showed that 71.53% ± 0.85% decolorization of CV (50 mg/L) was observed after 24 h. As compared to azo dyes, the triphenylmethane dyes are known to be resistant to enzymatic decolorization and hence require more time for dye decolorization [46]. Additionally, the dyes were not decolorized at the same extent that may be due to the difference of the redox potentials and the suitability of their steric structure with the active site of the enzyme [36]. In previous study, dye decolorization was found to be dependent upon the reaction time. When treated with peroxidases, 90% of Orange II was degraded during 36 h [47]. In this study, this laccase showed an excellent decolorizing capability, suggesting that it might be a promising candidate for dye decolorization.

### 2.5. Effect of pH, Temperature and ABTS Concentration on Decolorization Ability of Laccase

**Figure 5:**
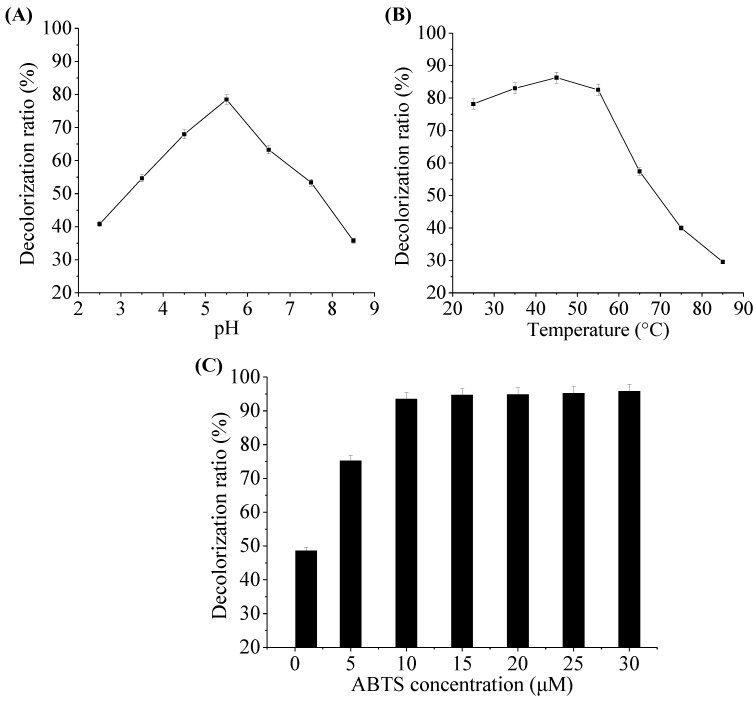
Effects of different parameters on MR decolorization in an ABTS-laccase mediated system: (**A**) pH; (**B**) Temperature; (**C**) ABTS concentration. Data were expressed as mean ± SD (n = 3).

Figure 5 shows the effects of pH, temperature, and ABTS concentration on MR decolorization by the laccase from *M. verrucaria* MD-R-16. The pH value played a key role in dye decolorization by laccase. As presented in Figure 5A, the laccase showed the decolorization of up to more than 63% in the broad range of pH (4.5–6.5), and the maximum MR dye decolorization occurred at pH 5.5. With pH above 5.5, the decolorization decreased due to the enzyme action. Temperature appears to affect enzymatic activities related to dye decolorization. As shown in Figure 5B, the increased MR decolorization was observed at a temperature range of 25–45 °C and reached the maximum decolorization at 45 °C. However, the decolorization decreased rapidly while temperatures were over 55 °C. As shown in Figure 5C, the MR decolorization ratio was correlated with the ABTS concentration. It was demonstrated that at least 10 μM ABTS was needed for more than 93% of dye decolorization within 24 h. When the ABTS concentration further increased, the MR decolorization efficiency was not obviously increased. ABTS as a redox mediator greatly enhanced dye decolorization, which was consistent with the result from Pan et al. [15], who reported that the indigo decolorization was increased from 2% to 75% by the addition of ABTS as the mediator. In this study, this laccase showed an excellent decolorization capability within broad range of pH and temperature, suggesting that it might be a relatively stable candidate for dye decolorization.

## 3. Materials and Methods

### 3.1. Chemicals and Reagents

2,2′-azino-bis(3-ethylbenzothazoline-6-sulfonate) (ABTS) was obtained from Sigma (St. Louis, MO, USA). Synthetic dyes (Table 3) and other reagents chemicals were of analytical reagent grade and purchased from Shanghai Chemical Reagents Company (Shanghai, China) and Tianjin Chemical Reagents Company (Tianjin, China), respectively. Buffers and solutions were prepared in distilled water.

### 3.2. Isolation and Culture of the Fungal Endophyte

Fungal endophytes were isolated from roots of pigeon pea [*C. cajan* (L.) Millsp.]. The fresh roots of pigeon pea were collected in the arboretum of the Key Laboratory of Forest Plant Ecology, Ministry of Education, Northeast Forestry University, Harbin, China. Roots were washed with running water to remove soil. The surface of the sample was sterilized. The samples were processed according to the method described by Zhao et al. [48], briefly in 75% ethanol (*v*/*v*) for 3 min, rinsed in sterile water and followed by 5% NaClO (*w*/*v*) for 7 min. Then, they were rinsed in sterile water three times and cut into 1 cm long segments. The surface-disinfected plant tissues were blotted dry on sterilized filter paper. Small tissue was placed in Petri dishes (9 cm in diameter) containing potato dextrose agar (PDA) medium. The dishes with plant tissues were cultured at 30 °C and examined daily for fungal growth until the mycelium appeared. Surface sterilization of uncut plant tissues served as effectiveness control. Furthermore, the fungal colonies were purified 3 times, numbered and transferred to fresh PDA slants separately. Finally, these slants were cultured for 7 days and then stored at 4 °C.

### 3.3. Screening of Laccase-Producing Fungal Endophyte

The isolated fungal endophytes from pigeon pea were cultivated for 7 days at 30 °C on PDA and then they were inoculated into Petri dishes on PDA containing 0.01% guaiacol (*w*/*v*). Guaiacol was a laccase indicator and added to the media before autoclaving [49]. Plates were cultured at 30 °C and examined daily for fungal growth until the positive halos of laccase-producing fungal endophytes were visible (reddish-brown in the presence of guaiacol) [24]. The ratio of colored halo size (HS) around the colonies to colony size (CS) was determined and used as an indicator to select suitable isolates for further investigation. Positive strains were subcultured on PDA with a laccase indicator or without, and they were aimed to confirm the presence of laccase in the selected fungal endophytes.

### 3.4. Identification of the Selected Fungal Endophyte by Morphological Properties and rDNA Sequence Analysis

The laccase-producing fungal endophyte was identified by the phylogenetic analysis of their ITS-rDNA sequences. According to the manufacturer′s instruction, the genomic DNA was extracted from the fungal cells using a Genomic DNA Extraction Kit (Tiangen Corp, Beijing, China). The total DNA was used as a template for PCR amplified using ITS1 (5′-TCCGTAGGTGAACCTGCG-3′) and ITS4 (5′-TCCTCCGCTTATTGATATGC-3′) primers specific fungal ITS-rDNA fragments [23]. The PCR mixture (50 μL) contained 25 μL of 2 × Taq Plus PCR Master Mix (Tiangen Corp.), 4 μL each of 10 μM primers, 2 μL of DNA template, and 15 μL of ddH_2_O. The PCR program amplification included an initial denaturation at 95 °C for 180 s, 30 cycles of denaturation at 94 °C for 30 s, annealing at 53 °C for 30 s, elongation at 72 °C for 60 s, and a final extension at 72 °C for 300 s. Amplified fragments were sequenced and purified. The sequencing of the PCR products were sequenced by Sangon Engineering Technology and Service Co. Ltd. (Shanghai, China). The sequencing result of fungal endophyte was submitted to GenBank and compared with known ITS-rRNA gene sequences in the GenBank database by BLAST [50]. Phylogenetic analysis was conducted using the neighbor-joining method with Molecular Evolutionary Genetics Analysis (MEGA) 4.0 software [51]. The NJ tree was assessed using pairwise genetic distances, which were based on all substitutions with the Jukes-Cantor distance parameter [52].

### 3.5. Laccase Production and Enzyme Preparation

For shake flask fermentation, the fungus *M. verrucaria* MD-R-16 was cultivated on PDA plate at 30 °C for 7 d and about five plugs with diameter of 5 mm were transferred into each 250 mL Erlenmeyer flask containing 100 mL seed medium. After cultivation at 30 °C and 120 rpm for 3 d, 3% (*v*/*v*) seed culture was inoculated into 50 mL basic nutrient medium and then incubated at 30 °C and 120 rpm for 5 d. The seed medium contained the following (per liter): glucose 20 g; peptone 5 g; yeast extract 3 g; KH_2_PO_4_ 3 g; and MgSO_4_·7H_2_O 1.5 g. The basic nutrient medium contained the following (per liter): potato 200 g; KH_2_PO_4_ 3 g; MgSO_4_·7H_2_O 1.5 g; VB_1_ 0.01 g; and CuSO_4_·5H_2_O 25 mg. Individual carbon source and nitrogen source were also added to the basal medium at the concentrations stated below. Various carbon supplements such as glucose, sucrose, maltose, lactose, and starch were tested to examine their effects on the laccase production. They were individually added at 2% (*w*/*v*) concentration to the basal medium (containing peptone 10 g/L as nitrogen source). Different organic and inorganic nitrogen supplements such as peptone, beef extract, yeast extract, ammonium nitrate, and ammonium chloride were tested to evaluate their effects on the laccase production. They were individually added at 1% (*w*/*v*) concentration to the basic nutrient medium (containing optimal carbon source at 20 g/L as carbon source). Moreover, to improve the laccase production, the fermentation conditions were optimized at different fermentation parameters such as fermentation time (1, 2, 3, 4, 5, 6, 7, 8, 9, and 10 d), temperature (24, 26, 28, 30, 32, 34, and 36 °C) and the initial pH (3.0, 4.0, 5.0, 6.0, 7.0, 8.0, and 9.0) on a rotary shaker at 120 rpm. For crude enzymes preparation, samples were withdrawn from the fermentation broth at 24 h intervals, and then filtrated through cotton gauze (four layers) followed by centrifugation at 10,000 rpm for 10 min to separate the cells from the medium. Crude enzymes were obtained from supernatants, stored at 4 °C and used for the enzymes assay.

### 3.6. Determination of the Enzyme Activity

The activity of laccase was determined by oxidation of ABTS as a substrate [24]. After incubation at 30 °C for 3 min, the increase in absorbance (due to oxidation of ABTS) was measured at 420 nm (*ε* = 36,000 M^−1^ cm^−1^) using UV-vis spectrophotometer (UNICO, Shanghai, China) [53]. The assay mixture (2 mL) consisted of 0.1 mL enzyme samples, 0.1 mL of 10 mmol/L ABTS, and 1.8 mL of 50 mmol/L sodium acetate buffer solution, pH 4.5. Lignin peroxidase (EC 1.11.1.14) activity and manganese peroxidase (EC1.11.1.13) activity were determined according to the method of Adak et al. [26] Bilirubin oxidase (EC.1.3.3.5) activity was determined as described by Murao et al. [29]. One unit of enzyme activity was defined as the amount of enzyme that can oxidize 1 μmol of substrate per minute under the assay condition. A reaction mixture without enzyme was also run.

### 3.7. Experimental Design

In order to achieve the efficient laccase production, a central composite design (CCD) combined with response surface methodology (RSM) was applied to optimize the laccase production by the fungal endophyte. Three variables, namely fermentation time (d), fermentation temperature (°C), and the initial pH, the three key factors related to laccase production, were selec ted as independent variables to investigate the effect on the laccase production. A total of 20 experimental sets were made to fit a full quadratic equation model, and six replicates at the center points were applied. The general equation was given below:

(2) $$Y=\beta_{0}+\sum_{j=1}^{k}\beta_{j}X_{j}+\sum_{j=1}^{k}\beta_{jj}X_{j}^{2}+\sum \sum_{i<j}\beta_{ij}X_{i}X_{j}\;(k=3)$$

where, *Y* represented dependent variable; *β*_0_, *β_j_*, *β_jj_*, and *β_ij_* were the regression coefficients for intercept, linearity, square, and interaction, respectively; *X_i_* and *X_j_* were the independent coded variables; and *k* represented the number of variables. The actual and coded levels of the independent variables used in the experimental design were shown in Table 1. The experimental design data and calculation of predicted responses were analyzed using Design-Expert 7.0 procedure (State-Ease, Inc., Minneapolis, MN, USA). Analysis of variance (ANOVA) was performed to calculate and simulate the optimal fermentation conditions of laccase production by the fungal endophyte.

### 3.8. Dye Decolorization

Four synthetic dyes, CR, MO, MR, and CV were used for investigating the efficacy of decolorization by laccase. Stock solutions (1 mg/mL) of these dyes were prepared in distilled water and diluted to the required concentration and then used for the decolorization assay. The reaction mixture contained citrate-phosphate buffer (0.1 M; pH 4.5), crude enzymes, and either CR (50 mg/L), MO (50 mg/L), MR (50 mg/L) or CV (50 mg/L) in a total volume of 2.5 mL. ABTS as a mediator was used to mediate the decolorization of dyes at the concentration of 0.5 mM as the stock solution. Then it was diluted to the required concentration for the decolorization assay. In all cases, the mixtures were incubated in a test tube in the dark at room temperature. The decolorization of structurally different dyes by laccase was monitored by the decrease in absorbance at the wavelength of each dye. The decolorization of tested dyes was calculated at different times (2, 4, 6, 8, 16, 18, 20, and 24 h). The decolorization ratio was calculated according to the following equation:

(3) $$\mathrm{Decolorization}\;\mathrm{ratio}\;(\%)=\left(A-A_{0}\right)/A_{0}\times 100\%$$

*A*_0_ and *A* represented the initial and final absorbance of the dye, respectively. A control test containing the same amount of the heat-denatured crude enzymes was also performed. To evaluate the effect of the pH (2.5–8.5) on MR (100 mg/L) decolorization, experiments were performed at the room temperature in the presence of ABTS (7 μM) with the same amount of crude enzymes. To evaluate the effect of the temperature (25–85 °C) on MR (100 mg/L) decolorization, experiments were performed at the pH 5.5 in the presence of ABTS (7 μM) with the same amount of crude enzymes. To evaluate the effect of ABTS concentration (1–25 μM) on MR (100 mg/L) decolorization, experiments were performed at the pH 5.5, and the temperature 45 °C in the presence of ABTS with the same amount of crude enzymes.

### 3.9. Statistical Data Analysis

All the experiments were performed in triplicate and the results were expressed by mean ± standard deviation (SD) (n = 3). The significant difference was calculated using a one-way ANOVA. Differences were considered significant when *p* was < 0.05.

## 4. Conclusions

In the present research, a novel fungal endophyte MD-R-16 capable of laccase-producing was firstly isolated from pigeon pea and its laccase possessed a strong ability for synthetic dye decolorization. Under optimal conditions, *M. verrucaria* MD-R-16 can obtain higher activity laccase production 16.52 ± 0.18 U/mL with a fermentation time of five days, temperature 30 °C, and pH 6.22. Moreover, the produced laccase could efficiently decolorize synthetic dyes within 24 h and be an excellent candidate for color removal of dyes within broad range of pH (4.5–6.5) and temperature (35–55 °C). Therefore, this novel laccase has great potential for the future application in industrial effluents. However, further research is required to purify the laccase, study the properties of the purified laccase, as well as its dye decolorization mechanism.

## Figures and Tables

**Table 1:** Results of the central composite design for the laccase production.

Runs	Factors			Laccase Activity (U/mL)	
	*X*_1_ (Time, d)	*X*_2_ (Temperature, °C)	*X*_3_ (pH)	Exp.^a^	Pred.^b^
1	−1 (2)	−1 (28)	−1 (5)	5.63	5.46
2	1 (6)	−1 (28)	−1 (5)	11.95	11.88
3	−1 (2)	1 (32)	−1 (5)	4.01	3.84
4	1 (6)	1 (32)	−1 (5)	8.03	7.86
5	−1 (2)	−1 (28)	1 (7)	6.44	6.30
6	1 (6)	−1 (28)	1 (7)	11.51	11.36
7	−1 (2)	1 (32)	1 (7)	7.59	7.32
8	1 (6)	1 (32)	1 (7)	10.12	9.98
9	−1.68 (0.64)	0 (30)	0 (6)	1.82	2.12
10	1.68 (7.36)	0 (30)	0 (6)	9.55	9.75
11	0 (4)	−1.68 (26.64)	0 (6)	11.68	11.88
12	0 (4)	1.68 (33.16)	0 (6)	9.05	9.36
13	0 (4)	0 (30)	−1.68 (4.32)	7.43	7.66
14	0 (4)	0 (30)	1.68 (7.68)	9.84	10.14
15	0 (4)	0 (30)	0 (6)	16.28	16.24
16	0 (4)	0 (30)	0 (6)	16.53	16.24
17	0 (4)	0 (30)	0 (6)	16.49	16.24
18	0 (4)	0 (30)	0 (6)	15.82	16.24
19	0 (4)	0 (30)	0 (6)	15.97	16.24
20	0 (4)	0 (30)	0 (6)	16.41	16.24

^a^ Exp. is expressed as experimental values. ^b^ Pred. is expressed as predicted values.

**Table 2:** ANOVA of the response surface quadratic model for the laccase production.

Source	Sum of Squares	DF	Mean Square	*F* Value	*p*-value Prob > *F*	Significance
Model	387.31	9	43.03	422.29	<0.0001	Significant
*X*_1_	70.10	1	70.10	688.17	<0.0001	
*X*_2_	7.62	1	7.62	74.84	<0.0001	
*X*_3_	7.46	1	7.46	73.23	<0.0001	
*X*_1_*X*_2_	2.93	1	2.93	28.75	0.0003	
*X*_1_*X*_3_	0.94	1	0.94	9.21	0.0126	
*X*_2_*X*_3_	3.51	1	3.51	34.47	0.0002	
*X*_1_^2^	191.68	1	191.68	1881.84	<0.0001	
*X*_2_^2^	57.21	1	57.21	561.64	<0.0001	
*X*_3_^2^	97.72	1	97.72	959.40	<0.0001	
Residual	1.02	10	0.10			
Lack of Fit	0.59	5	0.12	1.39	0.3726	Not significant
*R*^2^	0.9974					
Adjusted *R*^2^	0.9950					

**Table 3:** Characteristics of dyes used in this study.

Dye	Classification	Molecular Structure	Molecular Weight	λmax (nm)
Crystal Violet	Triphenylmethane	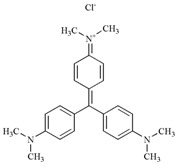	407.98	577
Congo Red	Azo	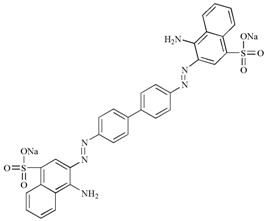	696.66	497
Methyl Orange	Azo	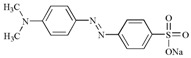	327.33	463
Methyl Red	Azo	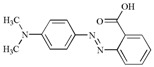	269.30	410
